# A Genome-Scale CRISPR Knock-Out Screen Identifies MicroRNA-5197-5p as a Promising Radiosensitive Biomarker in Colorectal Cancer

**DOI:** 10.3389/fonc.2021.696713

**Published:** 2021-07-30

**Authors:** Shijun Yu, Li Li, Kailing Fan, Yandong Li, Yong Gao

**Affiliations:** Department of Oncology, Shanghai East Hospital, Tongji University School of Medicine, Shanghai, China

**Keywords:** radioresistance, colorectal cancer, microRNA-5197-5p, CDK6, cell cycle

## Abstract

Radioresistance is one of the main reasons causing unsatisfactory curative effects of ionizing radiation (IR) against colorectal cancer (CRC). However, its underlying mechanisms remain unclear yet. In the present study, we applied a genome-scale CRISPR knockout screen in combination of NGS sequencing upon CRC cell lines to explore regulatory factors involved radioresistance of CRC, and 3 candidate genes were identified. Cytotoxicity of IR was determined by Cell Counting Kit-8 (CCK-8) assay, colony formation assay and apoptosis assay, and microRNA-5197-5p (miR-5197) was found to significantly enhance the cytotoxicity of IR to CRC cells. By further mechanistic investigation, we demonstrated that miR-5197 directly targeted CDK6 and inhibited its expression in RKO cells, which induced cell cycle arrest at G1/S phase and inhibited cell division, thereby radiosensitivity was enhanced by miR-5197. Our findings revealed that miR-5197 might be a critical factor regulating CRC cell radiosensitivity and provided novel insights into the development of therapeutic strategies for CRC patients who are resistant to IR.

## Introduction

Colorectal cancer (CRC) ranks the third most frequently diagnosed cancer and accounts for one of the leading causes of cancer-related death worldwide (1, 2). Apart from surgical resection and chemotherapy, adjuvant radiotherapy is another widely applied method in the treatment of CRC, which plays crucial roles in prolonging survival rates of patients with CRC (3, 4). However, approximately 50% patients are resistant to radiotherapy, which results in unsatisfactory curative effects, recurrence, and metastasis (5, 6). Therefore, it is necessary to ascertain underlying mechanisms of radioresistance to improve the therapeutic outcomes of CRC patients.

In the past decades, comprehensive approaches have been developed to identify elements that lead to CRC radioresistance (7–9). Although conventional loss/gain-of-function (such as RNAi and cDNA-based overexpression) methods have revealed some potential associations between specific genetic characteristics and response of CRC cells to radiotherapy (10), several limitations remain to be overcome. For example, RNAi-induced knockdown of genes is incomplete and nonpersistent, thereby hindering the identification of target genes that require complete inactivation.

With the emergence of clustered regularly interspaced short palindrome repeats (CRISPR) and CRISPR-associated protein 9 (Cas9) gene editing technology, utilizing functional genetic single-guide RNA (sgRNA) libraries to achieve more effective loss/gain-of function screening at genome level has become feasible (11, 12). For instance, Ji et al. identified kinases that modulate FGFR inhibitor response in gastric cancer using a functional CRISPR/Cas9 screen (13). In another cell culture model, genome-wide CRISPR-Cas9 knockout screen revealed SGOL1 as a druggable target of sorafenib-treated hepatocellular carcinoma (HCC) (14). Although these studies indicate the potential of CRISPR screen in evaluating the biological functions of genes involved in drug resistance, it is still unclear whether this approach is effective in identifying genes related with CRC radioresistance.

In the present study, we employed a genome-scale CRISPR knockout (GeCKO v2) library containing 123,411 targeting 19050 genes and 1864 microRNAs (miRNAs) sgRNAs (15) to systematically identify loss-of-function mutations conferring to radioresistance of CRC cells. Through NGS sequencing, miRNA-5197-5p (herein referred to as miR-5197) was identified as the most prominent candidate enhancing the killing effects of radiotherapy on CRC cells. Mechanistically, miR-5197 induced cell cycle arrest in G1/S phase and suppressed cell division by targeting Cyclin-Dependent Kinase 6 (CDK6) and inhibiting its expression.

## Materials and Methods

### Cell Culture and Reagents

Human CRC cell lines RKO, HCT116 and SW620 were purchased from the Shanghai Cell Bank of the Chinese Academy of Sciences (Shanghai, China). All cell lines were authenticated by short tandem repeat (STR) profiling analysis and cultured in Dulbecco’s Modified Eagle medium (DMEM, Corning, Inc., NY, USA) and RMPI-1640 medium supplemented with 10% fetal bovine serum (FBS, Corning, Inc., NY, USA) and 1% penicillin/streptomycin (M&C Gene Technology Ltd., Beijing, China) in a humidified incubator at 37°C with 5% CO_2_. The CDK4/6 inhibitor Palbociclib (PD-0332991) was purchased from Selleckchem, TX, USA, and the working solution was 500 nM.

### Ionizing Radiation (IR)

Cells were treated with IR by a 6-megavolt x-ray linear accelerator (Varian, EDGE, CA, USA) at different doses. The radiation conditions were as follows: treatment field: 40×40cm, source-skin distance: 100 cm, and radiation dose rate: 5 Gy/min.

### CRISPR Screen for Radioresistance of CRC Cells

The human GeCKOv2 CRISPR knockout pooled library was synthesized and purchased from Genechem Co, Ltd., Shanghai, China. Prior to the screening, the sgRNA library was sequenced by Genechem Co, Ltd., Shanghai, China to verify that all sgRNAs were presented. Briefly, RKO cells were transduced with the GeCKOv2 CRISPR knockout pooled library at a MOI of 0.3 and selected with puromycin for 1 week (2.5 μg/ml, Sigma, MO, USA). The transduced cells were divided into 3 groups with a minimum of 3 × 10^7^ cells for each and subjected to various doses of IR (0 Gy, 6 Gy and 12 Gy). After 1 week of culture, cells were rinsed with PBS and centrifuged to remove dead cells and debris, the surviving cells were harvested for genomic DNA extraction (Tiangen Biotech, Beijing, China). Then we amplified sgRNA region using PCR method and analyzed the enrichment of sgRNAs using NGS sequencing conducted by Oebiotech, Shanghai, China. The sequencing data after the screening has been deposited in the NCBI database Sequence Read Archive (SRA, accession number: PRJNA723460). All sgRNA reads and their targeting genes were shown, and nearly 90% of the intended targets were covered, indicating that the library was in good quality. Considering that enrichments of sgRNAs targeting specific genes in the surviving cells meant the absence of these genes promoted the radioresistance of RKO cells, sgRNAs with an increased copy number comparing with the control group (0 Gy) were considered as radiosensitivity candidate genes. The screening criteria was as follows: 1) log2 (fold change) ≥ 2.0; 2) false discovery rate (FDR)-corrected q-value < 0.05. 3) the copy number of candidate sgRNAs should be increased in both 6 Gy and 12 Gy groups compared to untreated cells.

### Transient Transfection

Small inference RNAs (siRNAs) against POLA2 (siPOLA2-1 and siPOLA2-2), RSAD2 (siRSAD2-1 and siRSAD2), miRNA-5197-5p mimics (miR-5197 mimics), miRNA-5197-5p inhibitor (anti-miR-5197), control siRNAs (siNC), control mimics (miR-NC) and control inhibitor were designed and synthesized by GenePharma Biotech (Shanghai, China). For transient transfection, the CRC cells were seeded into a 6-well plate at a density of ~40% per well. After cell attachment, cells were transfected with the indicated siRNAs or miRNA mimics/inhibitor using the Lipofectamine 3000 Reagents (Introgen, CA, USA) according to the manufacture’s protocol.

### Western Blot Analysis

To isolate cellular proteins, cells were lysed with RIPA lysis buffer (Beyotime, Jiangsu, China), protein concentration was quantified using a TaKaRa BCA Protein Assay Kit (Takara Bio, Kyoto, Japan). Standard western blot procedures were performed as described previously (16), and the immunoreactive signal was exposed by an Odyssey Infrared imaging system (LI-COR Biosciences, Lincoln, NE, USA). The specific primary antibodies used were as follows: anti-CDK6 (1:500, #14052-1-AP, ProteinTech, Wuhan, China), anti-Cyclin E2 (1:1000, #4132, Cell Signaling Technology, MA, USA) anti-β-actin (1:1,000, #81178, Santa Cruz Biotechnology, CA, USA).

### Quantitative Real-Time PCR (qRT-PCR) Analysis

Total RNA was extracted from CRC cells using TRIzol reagents (Sigma-Aldrich, Merck KGaA, Germany) and reverse transcribed into cDNA with a Primescript™ RT Reagent kit with gDNA Eraser (Takara, Kyoto, Japan) according to the manufacturers’ protocols. To measure miR-5197 levels, RNAs were reversely transcribed by specific stem-loop RT primer. Standard Quantitative real-time PCR procedures were conducted on an ABI QuantStudio™ 6 Flex system with SYBR-Green reagents (Takara, Kyoto, Japan). Data were analyzed by the comparative 2^ΔΔ^CT method. β-actin was selected as internal reference for POLA, RSAD2 and CDK6, and U6 was selected as internal reference for miR-5197. Primer sequences used were listed in **Table 1**.

**Table 1:** Primer sequences.

Name	Sequence (5’-3’)
CDK6-forward	GGATCTCTGGAGTGTTGGCT
CDK6-reverse	TGGTTGGGCAGATTTTGAATGA
POLA2-forward	TCTTCGGCCTAGACTGCGA
POLA2-reverse	CTATGCCTGGCTTTCGATAATCT
RSAD2-forward	TGGGTGCTTACACCTGCTG
RSAD2-reverse	GAAGTGATAGTTGACGCTGGTT
miR-5197-forward	GTGCAATGGCACAAACTCAT
miR-5197-reverse	GTGCAATGGCACAAACTCAT
miR-5197-stem loop primer	GTCGTATCCAGTGCAGGGTCCGAGG
	TATTCGCACTGGATACGACTCAAGA
β-actin-forward	CCTGGCACCCAGCACAATG
β-actin-reverse	GGGCCGGACTCGTCATACT
U6-forward	CTCGCTTCGGCAGCACA
U6-reverse	AACGCTTCACGAATTTGCGT

### Cytotoxicity Assay

Cell Counting Kit-8 (CCK-8) assays and colony formation assays were performed to determine CRC cell sensitivity to ionizing radiation. For CCK-8 assay, the indicated cells were seeded into a 96-well plate with a density of 3,000 cells/well and cultured for 24 h. At 48 h after treatment with ionizing radiation, cell viability was detected using a CCK-8 Kit (Dojindo, Kumamoto, Japan) in accordance with the instruction manual, and absorbance at 450 nm was measured on a microplate reader (SpectraMax M5, Molecular Devices, CA, USA). For colony formation assay, the indicated cells were plated into a 6-well plate at a density of 1,000 cells/well. After culture for 24 h, the cells were treated with ionizing radiation at the indicated doses and culture for 2 weeks. After staining with 0.5% crystal violet for 20 min, the colonies were photographed and counted.

### Flow Cytometry

Apoptosis and cell cycle distribution of CRC cells were evaluated by flow cytometry. For cell apoptotic rate analysis, the indicated cells were treated with radiotherapy at the indicated doses. After culture for 48, the cells were stained with the Annexin V‐FITC/PI Apoptosis Detection Kit (Dojindo, Kumamoto, Japan) and analyzed by flow cytometry (Arial II, BD Biosciences, CA, USA) following the manufacturer’s instruction manual. For cell cycle detection, the indicated cells were transfected with miR-5197 mimics/miR-NC for 48 h, and a cell cycle detection kit (Dojindo, Kumamoto, Japan) was used to measure cell cycle distributions on a CytoFLEX flow cytometer (Beckman Coulter, CA, USA), and data was analyzed using Modfit LT 5.0 software (Verity Software House, ME, USA).

### EdU Incorporation Analysis

Cell cycle phases and cell proliferation were evaluated using a Cell-Light EdU *In vitro* Kit (RiboBio, Guangzhou, China). Briefly, cells transfected with miR-5197 mimics/miR-NC for 24 h and seeded into a 12-well plate in exponential growth phase. After 24 h, the medium was changed to a medium containing EdU (μM) for 2 h at 37°C. After fixing the cells with 4% paraformaldehyde, Apollo and Hoechst 33342 staining was performed according to the manufacturer’s instructions. The EdU incorporation rates were quantified as the ratio of EdU-positive cells (red) to Hoechst 33342-positive cells (blue) under a fluorescence microscope (Leica Microsystems, Wetzlar, Germany).

### Dual Luciferase Reporter Assay

Wild-type (WT) and mutant (MUT-1/2/3) CDK6 3’UTR sequences with mutated miR-5197 binding sites were cloned into pGL3 luciferase reporter, respectively. The products were verified by DNA sequencing. Dual luciferase assays were performed using a Dual Luciferase Reporter Assay kit (Promega, WI, USA) following the operating manual. Briefly, the indicated cells were seeded into a 24-well plate in exponential growth phase. After transfection of miR-5197 mimics/miR-NC for 24 h, the cells were co-transfected with the indicated luciferase reporter constructs and pRL-SV40 plasmid containing the renilla luciferase gene. After 24 h, luciferase activities were quantified on a Dual-Glo^®^ Luciferase Assay System (Promega, WI, USA) following the specifications. Relative luciferase activity was calculated as the ratio of firefly to renilla luciferase activity.

### Statistical Analysis

Data were represented as mean ± standard error of the mean (SEM), and statistical analysis was performed using GraphPad Prism 7.0 software. Statistical significance of the data from independent experiments were determined using the two-tailed Student t-test or one-way analysis of variance (ANOVA), and P values < 0.05 were considered statistically significant.

## Results

### Identification of 3 Candidate Genes as Radiosensitivity Factors in CRC by CRISPR-Cas9 Knockout Screen

**Figure 1:**
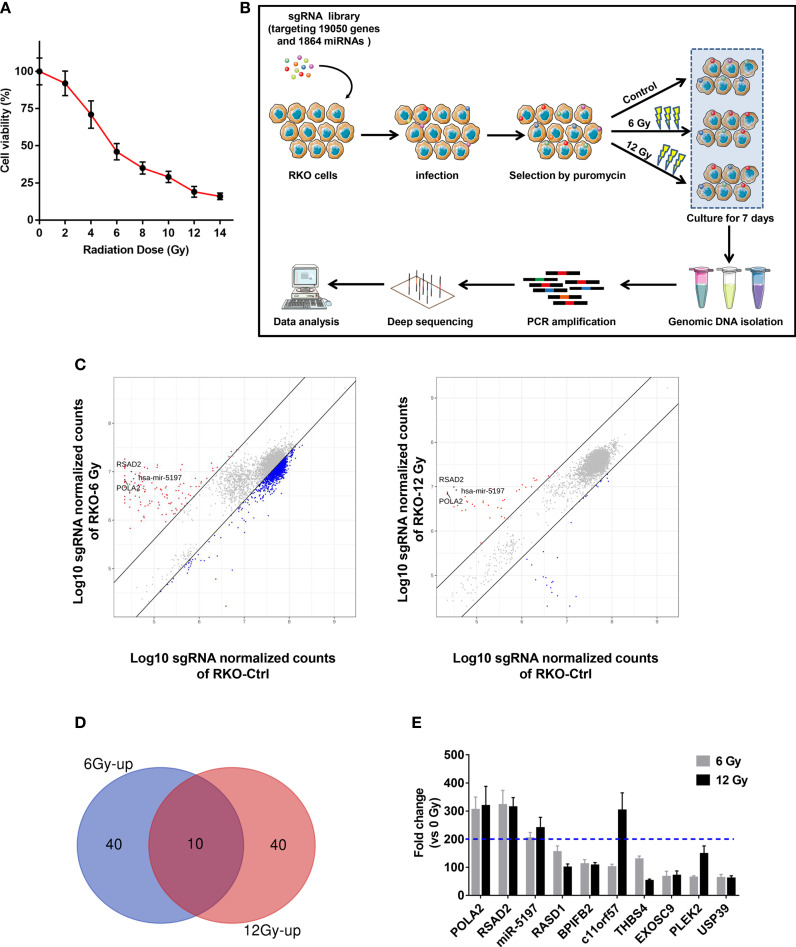
Identification of candidate genes related with CRC radioresistance by a genome wide CRISPR knock-out screen **(A)** RKO cells were treated with IR at the indicated doses, and CCK-8 assays were performed after 48 h. **(B)** A schematic picture showed the experimental setup of the screen. **(C)** Scatterplots of sgRNA frequency in RKO cells treated with 6 Gy (left) and 12 Gy (right) compared to the control group (0 Gy). **(D)** Venn diagram of the top 50 ranking sgRNAs up-regulated in 6 Gy and 12 Gy groups compared to the control group. **(E)** Top 10 ranking sgRNAs up-regulated in both 6 Gy and 12 Gy groups and their up-regulation fold changes were shown.

Prior to the screen, RKO cells were tested with ionizing radiation (IR) at different doses, and cell viability was evaluated to select the optimal dose for the screen. As shown in **Figure 1A**, the enhancement of viability inhibition became more distinct when the dose reached 6 Gy and became less evident when the dose was greater than 12 Gy. Therefore, 6 Gy and 12 Gy were selected as optimal doses. Then a high-throughput genome-scale CRISPR-Cas9 knockout screen was conducted as follows: RKO cells were transduced with a human GeCKO v2 library containing 123,411 sgRNAs targeting 19,050 genes and 1864 miRNAs, which is available from Addgene (http://www.addgene.org/crispr/libraries/geckov2/) and selected by IR (6 Gy or 12 Gy). After culture for 7 days, genomic DNA from the surviving cells was PCR-amplified and subjected to NGS sequencing (**Figure 1B**). Considering that enrichment of specific sgRNAs in the surviving cells meant knockout of their corresponding genes significantly promoted the resistance of CRC to IR, we focused on the up-regulated sgRNAs. According to the criterion adopted by this study, 190 and 77 increased sgRNAs were found in 6 Gy group and 12 Gy group, respectively (**Figure 1C**). To further narrow the possible candidate genes, we took the intersection of the top 50 up-regulated sgRNAs in 6 Gy group and 12 Gy group compared to the control group, and 10 candidate genes were finally identified (**Figures 1D, E**). Among these candidate genes, DNA polymerase alpha 2 (POLA2), radical S-adenosyl methionine domain containing 2 (RSAD2) and microRNA5197-5p (miR-5197) showed the highest fold changes in both 6 Gy and 12 Gy groups. Furthermore, qRT-PCR analysis also verified that POLA2/RSAD2/miR-5197 expression was significantly downregulated in the surviving cells (**Supplementary Figure 1**). Therefore, these 3 genes were subsequently selected as candidate radiosensitization factors of CRC.

### MiR-5197 Promotes CRC Cell Sensitivity to IR

**Figure 2:**
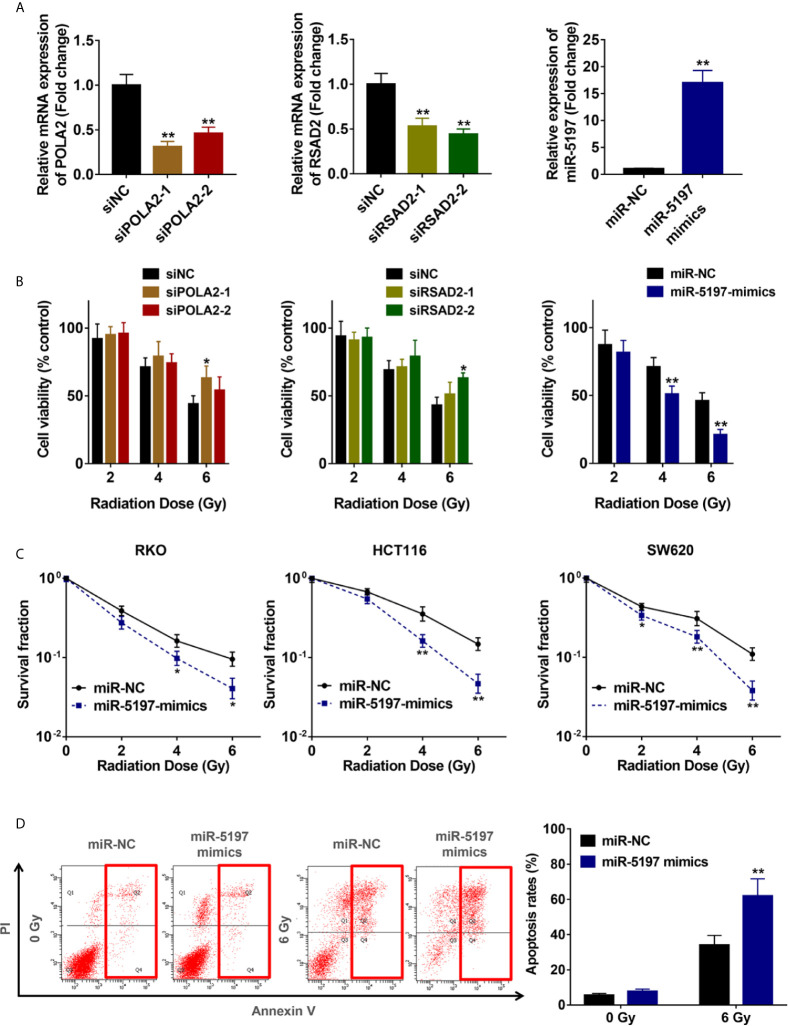
Mir-5197 was identified as a candidate radiosensitization factor in CRC cells **(A)** RKO cells were transfected with siRNAs against POLA2 and RSAD2 and miR-5197 mimics for 48 h, respectively. Then qRT-PCR analysis was performed to verify their efficiencies. **(B)** The indicated cells were treated with IR at different doses (0, 2, 4, 6 Gy), and CCK-8 assays were performed after 48 h. Cell viabilities were calculated according to the ratio of each group to the control group. **(C)** The indicated cells were used to performed colony formation assays to assess cell survival at 2 weeks after IR treatment. **(D)** Apoptosis rates of RKO cells after exposure to IR were examined by Annexin V/PI double-staining assays with flow cytometry. *P < 0.05, **P < 0.01.

To verify the accuracy of the screening results, expression of the 3 candidate genes in RKO cells were interfered by transfection with siRNAs against POLA2 and RSAD2 and miR-5197 mimics, respectively (**Figure 2A**). CCK-8 cell viability assays were performed after exposure to IR at different dosages (2/4/6 Gy). Knockdown of POLA2 or RSAD2 in RKO cells promoted cell radioresistance slightly, while transfection of miR-5197 mimics impaired radioresistance to a large extent (**Figure 2B**). Considering that cells with overexpression of miR-5197 showed the most prominent effect comparing with the other groups, we supposed miR-5197 might be an important factor in the radiosensitivity of CRC cells. Further clonogenic assays were performed after exposing different CRC cell lines to IR. Similar to the results of CCK-8 assays, miR-5197 overexpression significantly inhibited CRC cell viability after exposure to IR, and the inhibition was enhanced with increasing IR doses (**Figure 2C**). Additionally, we analyzed whether miR-5197 influenced cell apoptosis under different conditions (control *vs.* IR) by flow cytometry. Compared with the control group, miR-5197 overexpression strongly promoted IR-induced apoptosis of RKO cells (**Figure 2D**). Taken together, these data supported that CRISPR-Cas9 knockout screen is a potent tool for identification of genes involved in CRC radiosensitivity, and miR-5197 plays an important role in regulating CRC cell sensitivity to IR.

### CDK6 Is a Direct Target Gene of miR-5197

**Figure 3:**
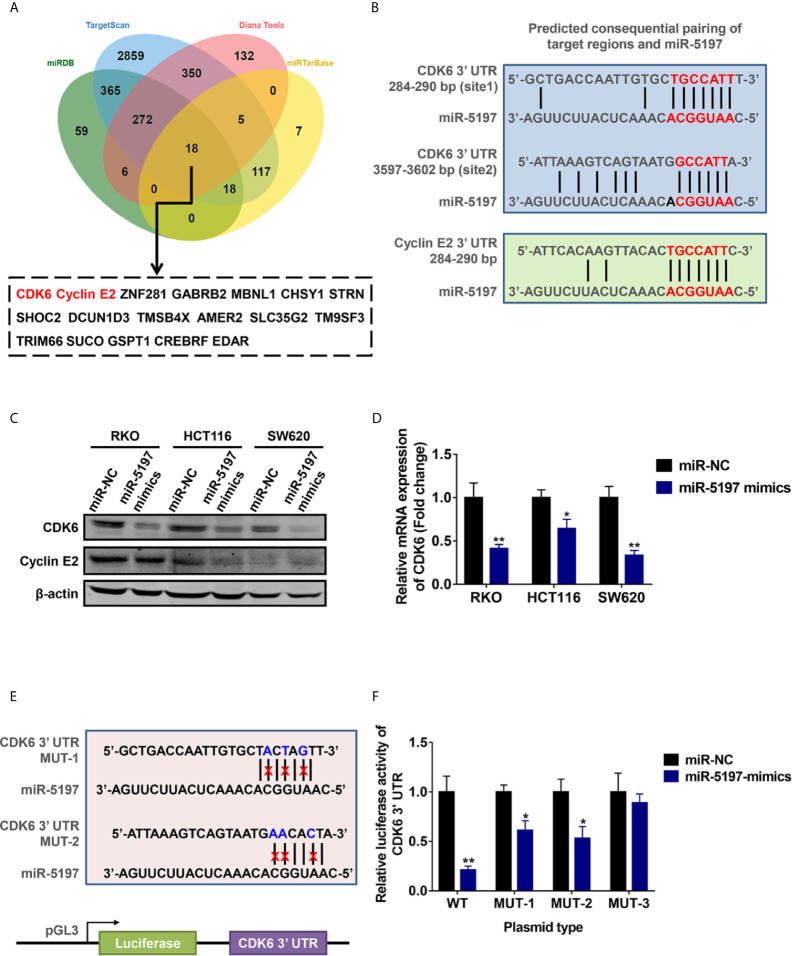
CDK6 was identified as a direct target of miR-5197 **(A)** 18 common genes were predicted could be targeted by miR-5197 using four databases. **(B)** Prediction of possible miR-5197 binding sites in the 3’UTR regions of CDK6 and CCNE2. **(C, D)** Western blot **(C)** and qRT-PCR **(D)** analyses were performed using RKO, HCT116 and SW620 cells transfected with miR-5197 mimics, respectively. **(E)** Wild type (WT) and mutant (MUT-1 and MUT-2) miR-5197 binding sites in the 3’-UTR of CDK6 were shown. **(F)** RKO cells with miR-5197 overexpression were transfected with the indicated reporter plasmids (MUT-3 contained mutation of both sites), dual luciferase reporter assays were conducted after 24 h. *P < 0.05, **P < 0.01.

To uncover the underlying mechanisms of miR-5197-mediated CRC sensitivity to IR, potential target genes of miR-5197 were predicted using 4 different miRNA binding prediction databases (miRDB, TargetScan, Diana Tools and miRTarbase), and 18 common target genes of miR-5197 were found (**Figure 3A**). Among them, CDK6 and cyclin E2 caught our attention, and putative miR-5197 binding sites in their 3’UTR regions were shown in **Figure 3B**. As we know, cell cycle regulation is crucial to cellular DNA damage response to IR, and both of CDK6 and cyclin E2 are essential regulatory factors that drive cell cycle transitions and their activities are under stringent control to ensure normal cell division (17–19), hinting us that CDK6 and cyclin E2 might be involved in the regulation of CRC radiosensitivity by miR-5197. Thus, we examined the effects of miR-5197 on CDK6 and cyclin E2 protein levels in different CRC cell lines. Overexpression of miR-5197 strongly inhibited CDK6 protein expression in RKO, HCT116 and SW620 cells, while no significant changes of cyclin E2 levels were observed between two groups (**Figure 3C**). Therefore, CDK6 was chosen for further experiments. Besides, we confirmed the suppressive effects of miR-5197 on CDK6 mRNA expression by qRT-PCR analysis (**Figure 3D**). To investigate whether miR-5197 could directly target CDK6, reporter plasmids containing wild type (WT) or mutant 3’UTR of CDK6 (MUT-1, MUT-2 or MUT-3) to perform dual luciferase reporter assays (**Figure 3E**). As expected, miR-5197 substantially inhibited the luciferase activity in RKO cells transfected with WT, which was largely weaken in cells transfected with MUT-1 and MUT-2, and this effect was almost completely absent in cells transfected with MUT-3 containing mutation of both miR-5197 binding sites (**Figure 3F**). The above findings demonstrated that miR-5197 could directly target CDK6 *via* specifically binding to its 3’UTR region.

### MiR-5197 Induces G1/S Cell Cycle Arrest and Retards Proliferation of CRC Cells

**Figure 4:**
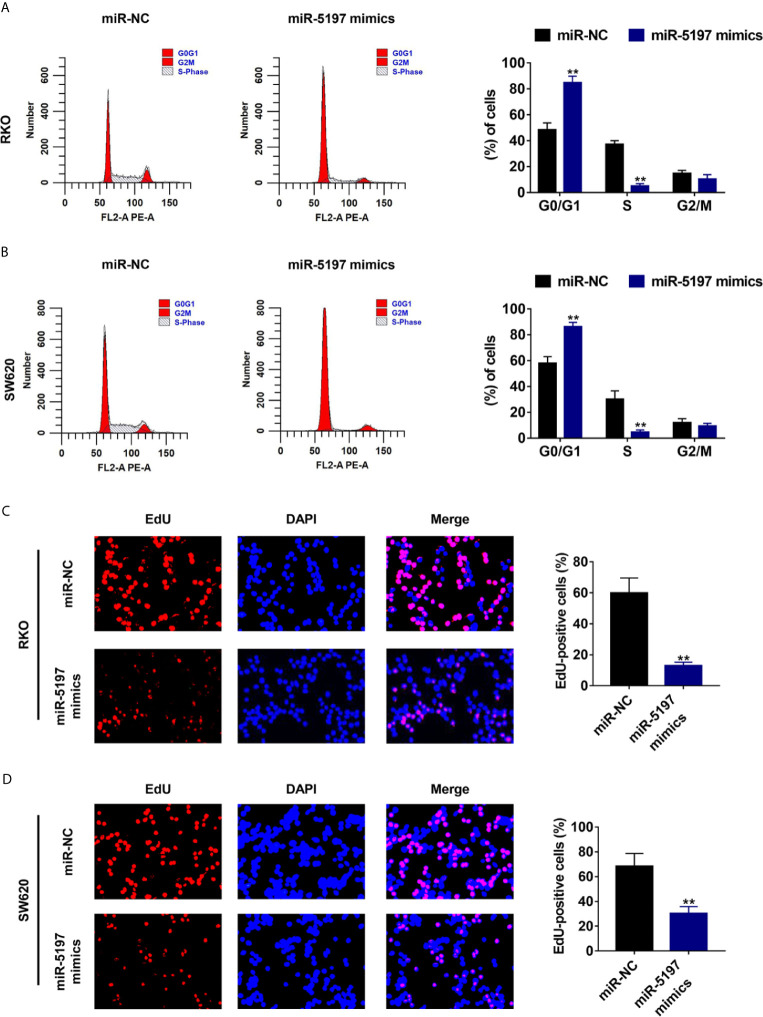
Mir-5197 induced cell cycle arrest at G1/S phase and inhibited CRC cell division **(A, B)** Cell cycle distribution of RKO **(A)** and SW620 **(B)** cells transfected with miR-5197 mimics was analyzed by flow cytometry (left panel), and proportion of each part was quantified (right panel). **(C, D)** Representative images (left panel) of EdU incorporation assays using RKO **(C)** and SW620 **(D)** cells with miR-5197 overexpression were shown. Cells undergoing DNA replication were stained with EdU (red) and cell nuclei were stained with DAPI (blue). The percentage of EdU-positive cells were quantified (right panel). **P < 0.01.

It has been well established that activated CDK6 forms a complex with CDK4 and cyclin D1 and initiates the phosphorylation of pRb releasing E2F transcription factors, thereby driving the expression of genes required for cellular commitment to enter S phase and ultimately mitotic cell division (20). Thus, we next examined whether miR-5197 influences cell cycle transition and cell division *via* inhibiting CDK6 expression. As evidenced by **Figures 4A, B**, G0/G1 portion in RKO and SW620 cells transfected with miR-5197 mimics increased largely compared to the control cells. In contrast, a sharp reduction of S portion was observed after miR-5197 overexpression. EdU incorporation assays were then carried out to explore whether cell division is altered by miR-5197. Compared with the control group, the ratio of EdU-positive cells to total DAPI-positive cells was remarkedly decreased in RKO and SW620 cells with miR-5197 overexpression (**Figures 4C, D**). Thus, these data verified that miR-5197 induced G1/S cell cycle arrest and inhibited CRC cell division by targeting CDK6.

### Radiosensitization by miR-5197 Is Mediated by Targeting CDK6

**Figure 5:**
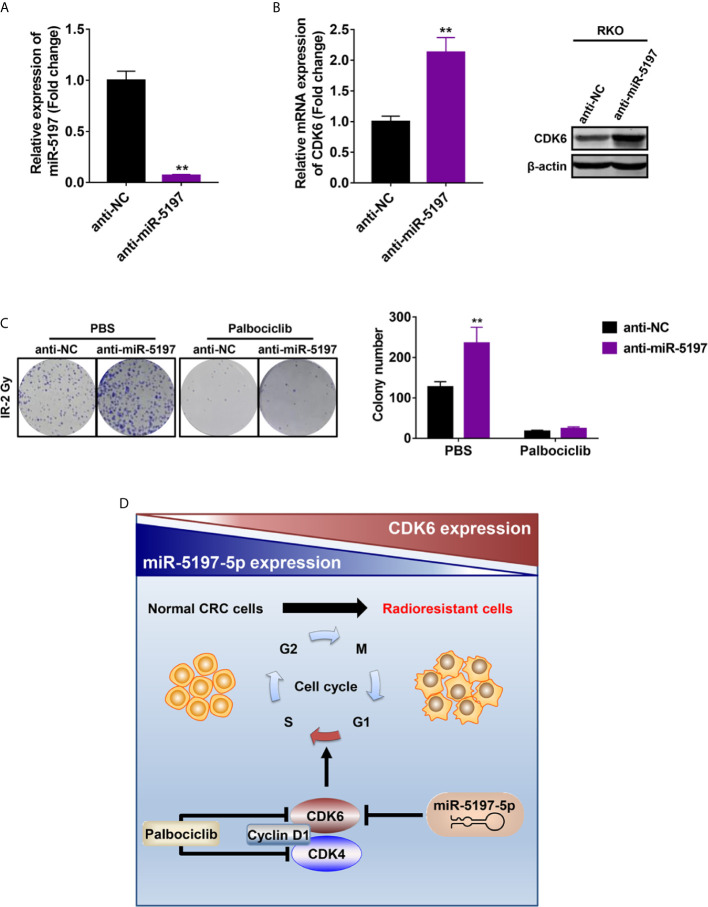
Mir-5197 promoted CRC cell sensitivity to IR by regulating the expression of CDK6 **(A)** RKO cells were transfected with miR-5197 inhibitor (anti-miR-5197) and its efficiency was verified by qRT-PCR analysis. **(B)** CDK6 mRNA (left) and protein (right) expression in RKO cells transfected with anti-miR-5197 were analyzed by qRT-PCR and western blot analyses, respectively. **(C)** RKO cells with knockdown of miR-5197 were exposed to IR (2 Gy) after treatment with Palbociclib (500 nM) or PBS, and colony formation assays were performed. Representative images (left) and quantification (right) of colonies were shown. **(D)** A schematic overview of the mechanism underlying miR-5197-mediated radiosensitization of CRC cells. In normal CRC cells, miR-5197 targets and inhibits CDK6 expression, thereby inducing cell cycle arrest and enhancing the sensitivity to IR. In radioresistant CRC cells, with lower miR-5197 expression, CDK6 level is up-regulated, and cells are more sensitive to Palbociclib. **P < 0.01.

Previous studies have revealed that CDK6 enhances radioresistance in human malignancies including nasopharyngeal cancer and HPV negative head and neck squamous cell carcinoma possibly by promoting RAD51 and BRCA1 expression and thereby activating homologous recombinational (HR) DNA repair after IR (21, 22). Given the regulatory effects of miR-5197 on CDK6 expression, we next asked whether miR-5197 repressed CRC radioresistance by inhibiting CDK6 expression. After transfection of miR-5197 inhibitor (anti-miR-5197) or negative control miRNA inhibitor (anti-NC) into RKO cells for 48 h (**Figure 5A**), qRT-PCR and western blot analyses confirmed the positive effects of miR-5197 inhibition on CDK6 mRNA and protein levels, respectively (**Figure 5B**). Subsequently, these cells were treated with Palbociclib (PD-0332991), a highly selective CDK4/6 inhibitor, and colony formation assays were conducted after exposure to IR (2 Gy). Down-regulation of miR-5197 significantly promoted radioresistance of RKO cells. In contrast, cells treated with Palbociclib alone were more sensitive to IR, which was consistent with previous studies. More importantly, the effects induced by anti-miR-5197 were completely reversed by Palbociblib treatment (**Figure 5C**). Therefore, these findings provided strong credence to our hypothesis that miR-5197 promoted CRC cell radioresistance *via* targeting CDK6.

## Discussion

Numerous studies have revealed that radioresistance is closely associated with recurrence after radiotherapy and worse prognosis of CRC patients (5, 8). To date, however, no such clear predictive biomarkers have been discovered to guide IR treatment. In the present study, we performed a genome-wide CRISPR-Cas9 knockout screen in combination with NGS sequencing to systematically identify loss-of-function mutations conferring to radioresistance of CRC cells for the first time. Our data showed that miR-5197 contributes to enhanced sensitivity of CRC cells to IR and may serves as an effective biomarker to select CRC patients who could benefit the most from IR treatment.

Mir-5197, located on the long arm of chromosome 5, was initially identified in a study of pediatric acute lymphoblastic leukemia (ALL) through high-throughput sequencing (23). It has been reported that the polymorphisms of miR-5197 contributes to the chemotherapy toxicity of lung cancer (24). However, its biological functions in cancers including CRC, is still unclear. Herein, we found that sgRNA specifically targeting miR-5197 was enriched in surviving RKO cells after exposure to 6 Gy and 12 Gy of IR, which suggested that its deficiency enhanced resistance of CRC cells to IR. Further CCK-8 and colony formation assays confirmed that miR-5197 significantly weakened CRC cell viability and promoted cell apoptosis after exposure to IR. These findings indicated that radioresistance of CRC patients could be due to down-regulation of miR-5197 expression in tumor cells. Nonetheless, it is necessary to verify our results using an *in vivo* radiotherapy model in future studies.

Mechanistically, CDK6, an important cell cycle regulatory protein, was identified as a direct target of miR-5197. By inhibiting CDK6 mRNA and protein expression in CRC cells, miR-5197 induced cell cycle arrest in G1/S phase and retarded cell division. Additionally, treatment with Palbociclib (a highly selective inhibitor of CDK4/6) reversed the positive effects on radioresistance induced by down-regulation of miR-5197, hinting us that miR-5197 promoted CRC cell sensitivity to IR *via* targeting CDK6.

As one of the most important cell cycle machineries, CDK6 forms an active complex with CDK4 and cyclin D1 to phosphorylate downstream proteins such as the retinoblastoma tumor suppressor protein (RB) and E2F transcription factors, activation of which finally promotes cell cycle progression through G1 to S phase (20, 25). Palbociclib is the first selective CDK4/6 inhibitor applied in the treatment of breast cancer (26, 27), and it has been reported to enhance radiosensitivity of HPV negative head and neck squamous cell carcinomas by inhibiting CDK4/6 (21). Our findings provided further evidence in support of this conclusion. From a clinical perspective, it is possible that patients with lower miR-5197 expression will benefit more from combination therapy (Palbociclib + IR) than IR alone.

In conclusion, the present study identified miR-5197 as a radiosensitization factor that promoted IR-induced apoptosis of CRC cells. Mechanistically, miR-5197 inhibited CDK6 expression and thereby promoting cell cycle arrest in G1/S phase and inhibiting cell division (**Figure 5D**). Our findings might provide novel insights into the development of precise therapeutic strategies against CRC in the future.

## Data Availability Statement

The datasets presented in this study can be found in online repositories. The names of the repository/repositories and accession number(s) can be found below: NCBI database Sequence Read Archive (SRA), accession number: PRJNA723460 (https://www.ncbi.nlm.nih.gov/sra/PRJNA723460).

## Author Contributions

YG and YL designed this study. SY, LL, and KF performed experiments and statistical analysis. SY and LL participated in writing and revising the manuscript. YG and YL reviewed the data and manuscript. All authors contributed to the article and approved the submitted version.

## Funding

This study was supported by grants from the National Natural Science Foundation of China (No. 81972290) and the Outstanding Clinical Discipline Project of Shanghai Pudong (No. PWYgy2018-02). The funding sponsor reviewed and approved the study protocol, and the final version of the manuscript. All analytical decisions were made by the authors, and the final version of the manuscript was approved by all authors.

## Conflict of Interest

The authors declare that the research was conducted in the absence of any commercial or financial relationships that could be construed as a potential conflict of interest.

## Publisher’s Note

All claims expressed in this article are solely those of the authors and do not necessarily represent those of their affiliated organizations, or those of the publisher, the editors and the reviewers. Any product that may be evaluated in this article, or claim that may be made by its manufacturer, is not guaranteed or endorsed by the publisher.

## Supplementary Material

Supplementary Figure 1 The mRNA expression levels of POLA2/RSAD2/miR-5197 in surviving cells after CRISPR-Cas9 knockout screen were examined by qRT-PCR analysis. **P < 0.01.
