# Supplementary figures and images for: A Genome-Scale CRISPR Knock-Out Screen Identifies MicroRNA-5197-5p as a Promising Radiosensitive Biomarker in Colorectal Cancer
Source: Front Oncol. 2021 Jul 30;11:696713. doi: 10.3389/fonc.2021.696713 (PMC8362832; doi:10.3389/fonc.2021.696713)

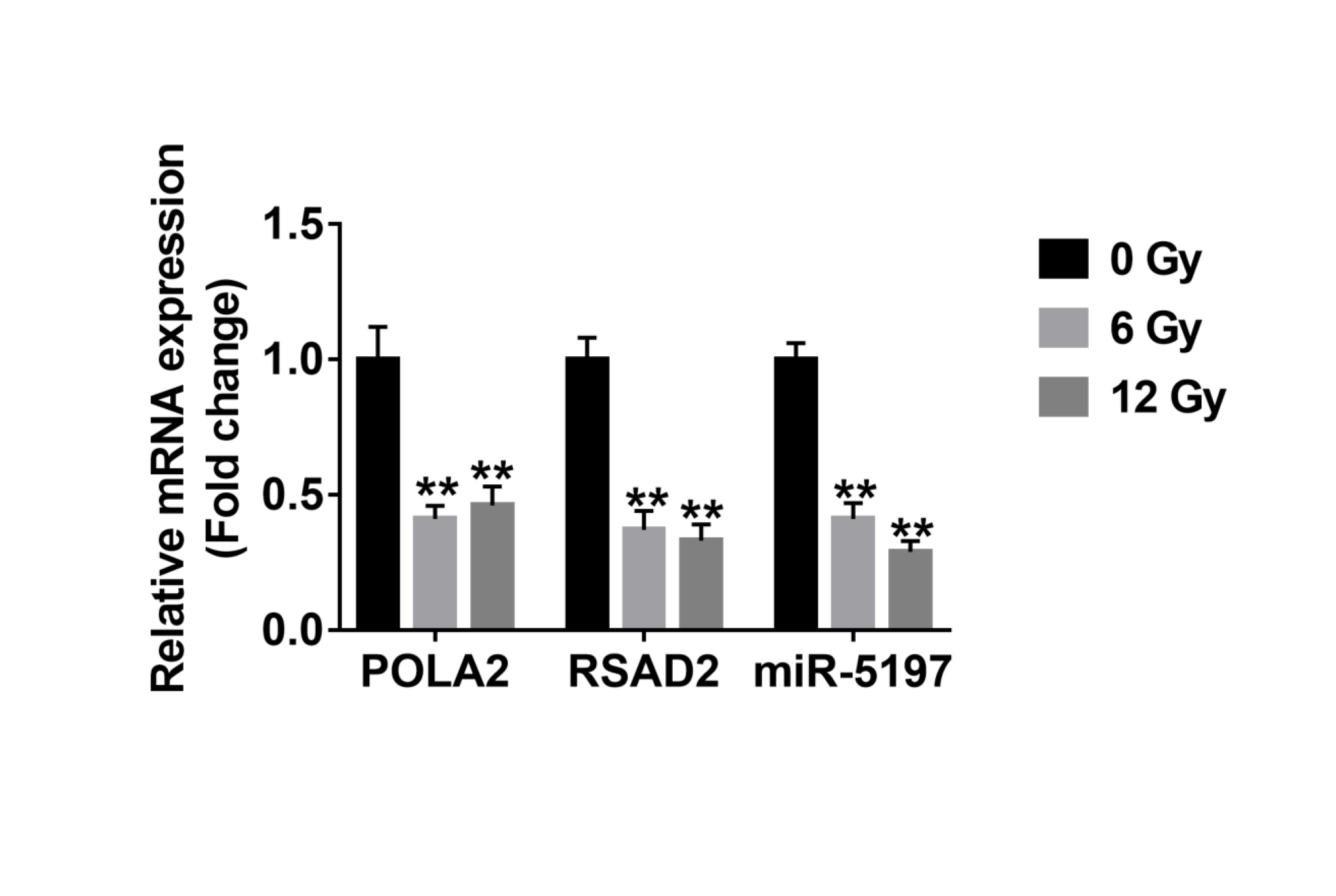

Supplement: Supplementary Figure 1 — The mRNA expression levels of POLA2/RSAD2/miR-5197 in surviving cells after CRISPR-Cas9 knockout screen were examined by qRT-PCR analysis. **P < 0.01. [file Image_1.tif]
